# Sustainable development goals for industry, innovation, and infrastructure: demolition waste incorporated with nanoplastic waste enhanced the physicomechanical properties of white cement paste composites

**DOI:** 10.1007/s13204-023-02766-w

**Published:** 2023-01-25

**Authors:** M. A. Abdelzaher

**Affiliations:** Environmental Science and Industrial Development Department, Faculty of Postgraduate Studies for Advanced Sciences, Beni-Suef University, Beni Suef, 62511 Egypt

**Keywords:** COVID-19 pandemic, Nano-plastic-waste (NPW), Ultra-fine demolition waste (UDW), Eco-white cement (E-WC), Energy saving, Whiteness reflection (Ry), Sustainability

## Abstract

The COVID-19 pandemic significantly impacts the increase in plastic waste from food packaging, masks, gloves, and personal protective equipment (PPE), resulting in an environmental disaster, if collected, processed, transported, or disposed inappropriately. Plastic waste has a very long deterioration time in the environment (soil and water), cheap, and plentiful. Additionally, construction waste disposal is a process that transfers debris to a state that does lead to any sustainable or environmental problems. The core objective of this current research work is to provide safety and efficacy by partial substitution of both ultrafine demolition waste (UDW), incorporated with nanoplastic waste (NPW), for eco-white cement (E-WC) composition. E-WC is designed by partially substituted WC with UDW (1.0, 5.0, 10.0, 15.0, and 20.0 wt.%); incorporated with NPW (1.0 and 3.0 wt.%); to adequately protect people and the environment over long periods. The context examines the high performance, physicomechanical properties and high durability of blends as presences of silica in UDW proposed a hydraulic filler material, plus; high surface area of NPW. The microstructure and workability are characterized by X-Ray Fluorescence (XRF), Scanning Electron Microscope (SEM), and Transmission Electron Microscope (TEM) measurements. The record results show greatly enhanced in the mechanical strength due to the combination of NPW and UDW (active silica). With the presence of NPW and UDW in WC matrix, the highest level of crystallization formed consequently a decrease in whiteness reflection (Ry) and total porosity. In summary, WC blend with NPW and UDW reflects better workability and energy saving qualities, which are economical and environmentally beneficial and may result in decreased construction budget and improve a long-term raw material sustainability.

## Introduction

**Fig. 1:**
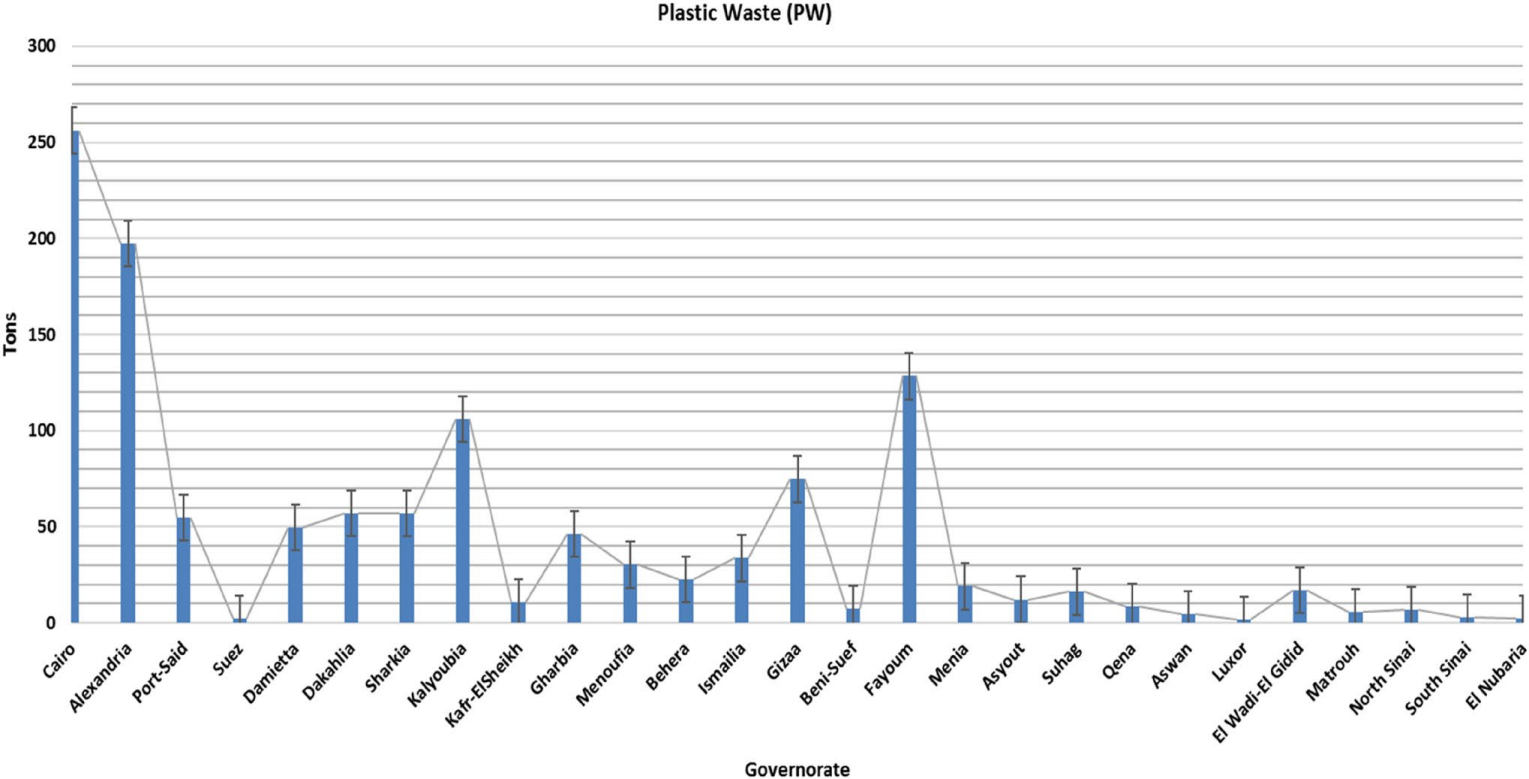
Production of plastic waste (Tons) in the governorates of Egypt

Plastic waste (PW) is present in the surrounding environment with less use for recycling or as a substitute for industrial raw materials. There are seven types of plastic, all of which are harmful except for the fifth type (BPA free). Modern waste management techniques reduce the rapid depletion of both resources, including raw and combustible materials. Solid waste has significant negative drawbacks to the ecosystem and living conditions (Mukherjee et al. 2021; Adarsh et al. 2022; Saleh et al. 2021; Singh and Sharma 2016). Additionally, a large portion of plastic waste appears with increasing the production scale, where plastic solid waste negatively affects the environment. Many local and international rules have emphasized the need to examine waste recycling and landfilling to minimize its negative impacts. A review of the literature indicates that many investigations have focused on plastic waste to reach more environmental efficiency and applicable materials (Evode et al. 2021; Gupta et al. 2019). The huge increase in the popularity of using environmentally friendly, low-cost, and dangerous materials in the productivity of building materials has led to the need for a deep investigation of how to achieve this on a large scale using the environment as well as preserving the materials and confirming the requirements within acceptable limits according to cement and concrete specs (Goli et al. 2020). Particularly, in the last 5 years, the usage of plastic in food packing and PPE has hugely increased. Thus, with an increase in the production of plastic factories, the volume increases and the PW cannot be stored or recycled in the conventional ways (e.g., landfilling and/or burning) (Owaid et al. 2022; Osial et al. 2022; Salih et al. 2022). In 2025, the production of plastic waste may reach 21 BT (Ncube et al. 2021; Hahladakis et al. 2020). Figure 1 shows the production of plastic waste (tons) in the governorates of Egypt, which was assumed to be ~ 3603.81 tons according to the Egyptian mobilization and statistics center in the period from 2018 to 2021(Abdelzaher et al. 2018). The implementation of this industrial waste that cannot be stored in other sectors and economic gains can be made from them while reducing environmental pollution (Barnes 2019). Inorganic and economic materials, e.g., supplementary cementitious materials (SCMs), have remarkable increasing potential in advanced industries, such as paper, agriculture, organic fertilizer, glass, chemicals, and construction material industries. In addition to; PW may be helpful raw material due to their low cost and availability (Shahani et al. 2021; Abbas et al. 2021; Zalasiewicz et al. 2019).

The disposal of demolition debris waste is one of the most important sectors for which innovative solutions are necessary, in unconventional ways, especially since the accumulation of demolition and construction waste constitutes a real and growing environmental problem (Marzouk and Azab 2014; El-Kattan et al. 2020; Benjeddou et al. 2020). The recycling of construction waste is an area of ​​ potential for engineers, as the volume of accumulated waste in Egypt is around 50 million tons, in addition to 5 million tons annually (Barnes 2019; Benjeddou et al. 2020). Construction and demolition waste represents about 44% of it, and no companies recycle construction waste in Egypt, except for one company that uses shredding equipment for paving roads and streets, although there are about 66 garbage-sorting plants. It is obligatory to enhance integrated and sustainable solutions for managing construction waste to preserve material resources such as minerals and ores. Increasing the resource productivity, and improving the reuse and recycling of materials in a way that reduces the depletion of raw resources, preserves the environment and contributes to achieving development and environmental sustainability goals in Egypt (Kineber et al. 2020; Abdelzaher et al. 2018; Nik and Bahari 2012; Balboul et al. 2019; Abdelzaher and Shehata 2022). The operation includes the collection, transportation, sorting and recycling of waste emitted from construction and demolition works, benefiting from building materials, recycling and reusing them at the project site to reduce transportation costs, dispose of waste, preserve natural resources, and benefit in a manner that achieves the requirements of the leadership system in energy and environmental designs. Nanotechnology has a wonderful approach and various applications in the cement and concrete fields. Enhancing the physical–mechanical and chemical properties of the WC microstructure when incorporated with nanoparticles is attributed to the multi-different uses of nanoparticles because of their unique properties. Many research works on adding different nanomaterials to cement and concrete have been reported elsewhere (Du et al. 2019; Tantawy et al. 2013; Kong et al. 2018). Physic-mechanical and chemical processes of cement hydration process are complicated (Kong et al. 2018; Bellmann et al. 2010; Scrivener et al. 2019). The topo-chemical conventional theory and through reactions of the solution are complex mechanisms, which deeply explain the C–S–H gel formation once it starts the hydration process in an advanced way (Scrivener and Nonat 2011; Scrivener et al. 2015; Ludwig and Zhang 2015). Nano-material applications are limited due to financial issues. These materials have self-cleaning properties that trigger the photocatalytic degradation of most pollutants in the air (Schneider 2015). Additionally, high Blaine (surface area) acts as active nuclei during cement hydration, and promotes the formation of C–S–H and C–A–H phases (Du et al. 2019; Abdelzaher 2022; Sobolev 2016; Silvestre et al. 2016).

However, the implementation of NPW incorporated with ultrafine-UDW as a supplementary material for white cement fabrication not investigated. The main objective of this practical study was to investigate the effectiveness of PW incorporated with ultrafine-UDW used at various replacement levels on the properties of WC in terms of compressive mechanical strengths, whiteness reflection (Ry), porosity, and microstructure.

## Laboratory program

### Materials

**Fig. 2:**
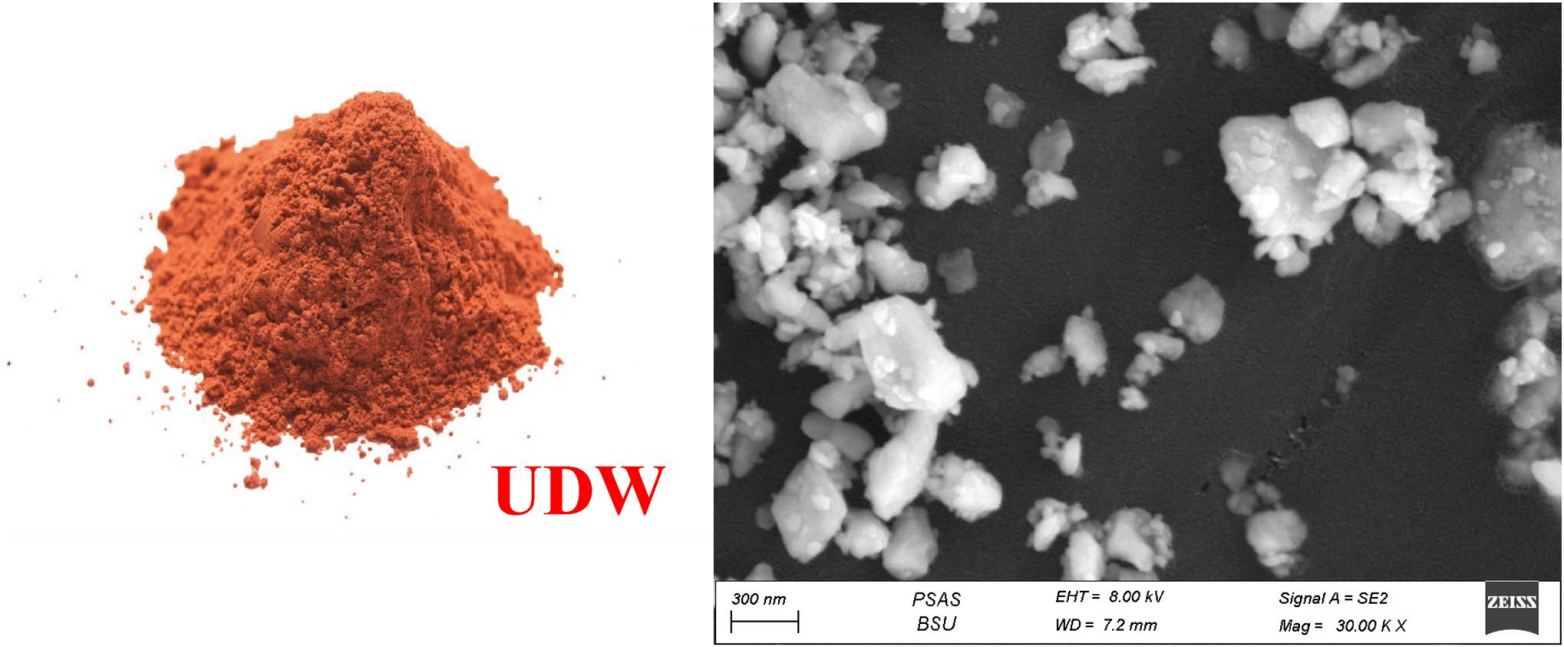
UDW visually inspection  and SEM microstructure

**Fig. 3:**
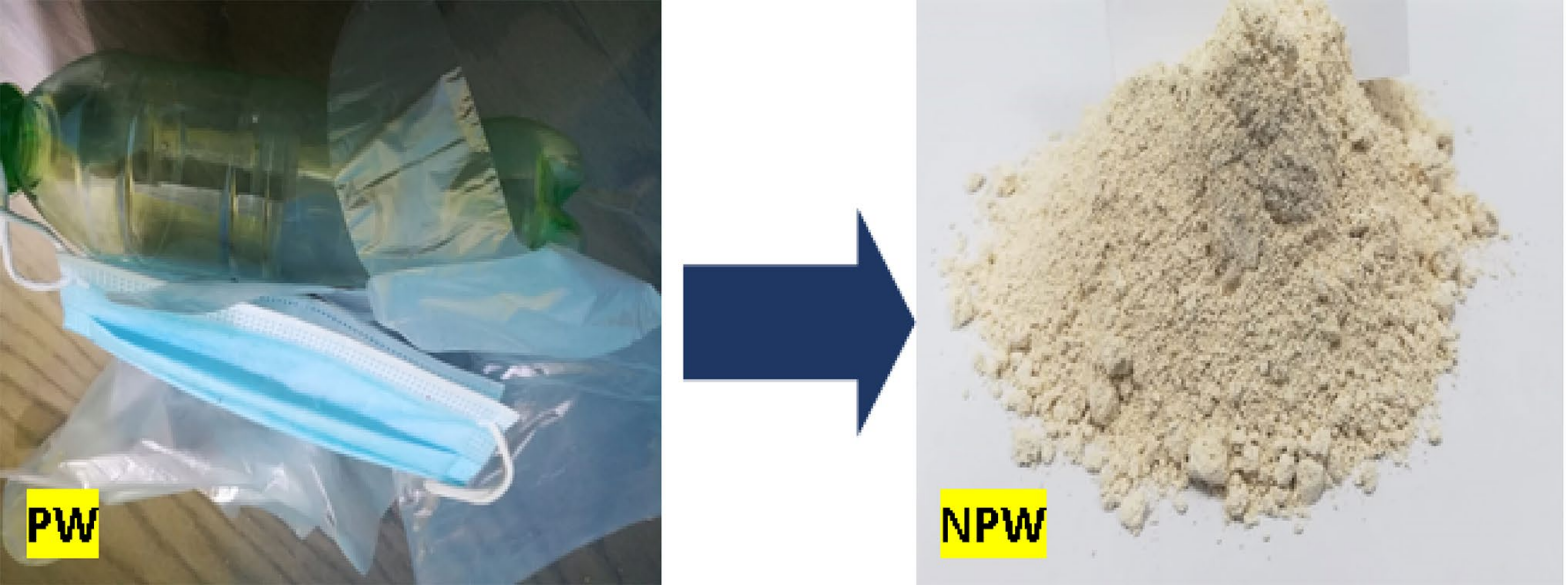
Visual inspection of PW and NPW

**Fig. 4:**
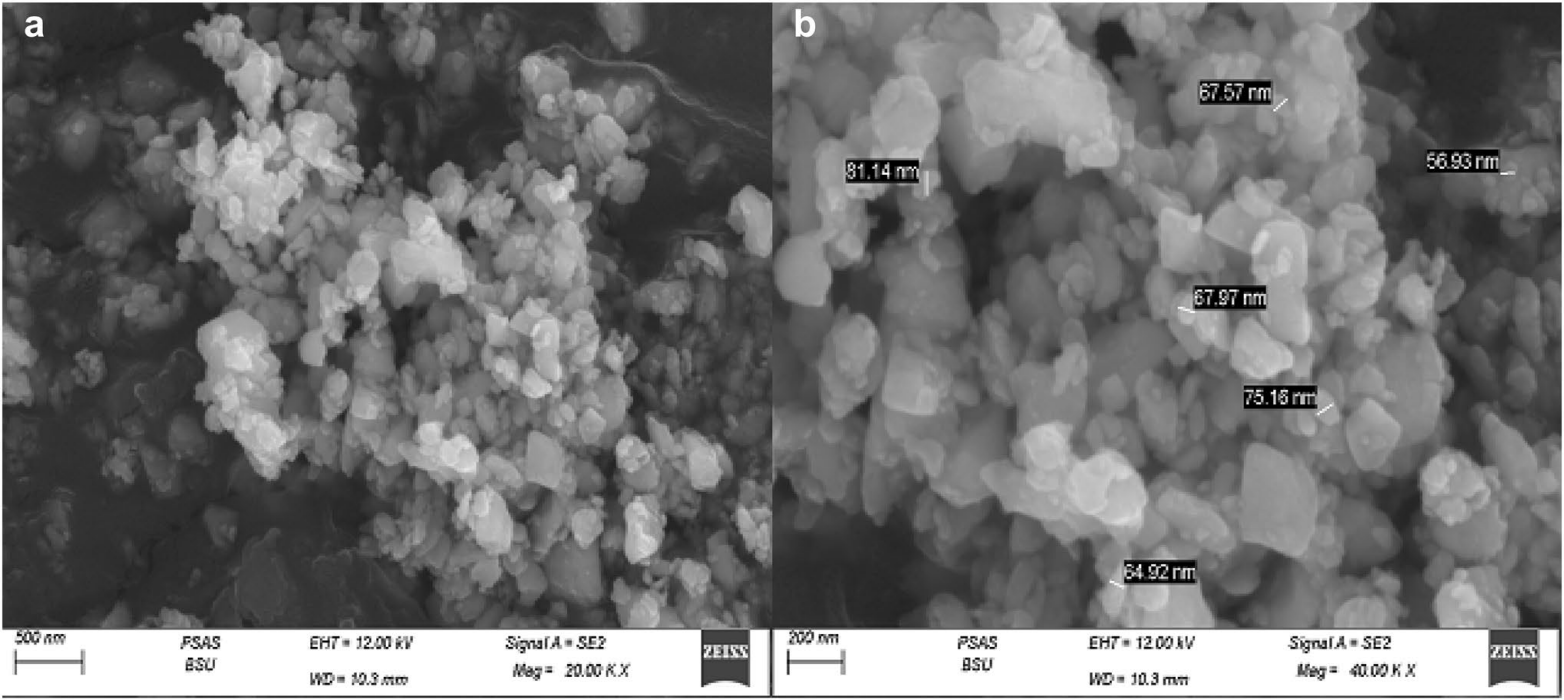
**a**, **b** SEM and TEM photos of NPW

In the current lab program, white cement (WC), ultrafine demolition debris waste (UDW), and nano-plastic-waste (NPW) represent the core materials. White cement [Class I, 52.5 MPa] was purchased from Sinai White Cement Company (SWCo.), (Sinai, Egypt). Demolition debris waste was brought from Tourah area, (Giza, Egypt). Crushing then milling until reach ultrafine size passes through 63-µm mesh, specific surface area is, e.g., Blaine 4199 cm^2^ g^−1^. Figure 2, shows the UDW visually and SEM microstructure. Nano-plastic waste processed by cutting the PW (bags + pottiles + PPE) into small sizes as possible, as shown visually in Fig. 3. Small pieces of PW were grained in the ball mill for 3 days (continuously). Powder is checked every 12 h by sieving it on 63 µm mesh till reach nanosize. Figure 4a, b shows the SEM and TEM morphologies of NPW, respectively, and prove that PW has skeleton arrangement particles and reaches the nanosize scale, e.g., NPW size between 56.93 and 81.14 nm. Detailed XRF analysis for white cement and ultrafine demolition debris waste, e.g., the chemical analysis for WC, is shown in Table 1.

**Table 1:** XRF analysis of white cement and ultrafine demolition debris waste

Elements	SiO_2_	Al_2_O_3_	CaO	Fe_2_O_3_	MgO	SO_3_	Na_2_O	K_2_O	LOI	Cl^−^
WC	22.31	2.92	67.46	0.11	0.26	2.90	0.05	0.05	2.61	0.06
UDW	68.11	16.84	7.03	0.12	1.44	0.02	0.87	1.05	1.98	0.03

### Preparation and testing methods

Generally, two sets of works are proposed. WC was substituted with various portions, e.g., (1.0%, 5.0%, 10.0%, 15.0%, and 20.0) by wt. % UDW, in the presence of 1.0% and 3.0% NW by wt.% (fixed ratio), and all proposed sets are tabulated in Table 2. Additionally, water/cement powder ratio (W/CP) was reported for all M_*x*_G_*x*_ composites.

**Table 2:** Mix composition of proposed blends (as replacement)

Blend composition	White cement batch by weight	Nano-plastic-waste batch by weight	Ultra-fine demolition debris waste	Total mix batch by weight	Water/cement ratio by weight
M0 (Ref.)	100.00	0.00	0.00	100.00	0.28
Group I: NPW ratio 1.0 wt. %					
M1G1	98.00	1.00	1.00	100.00	0.36
M2G1	94.00	1.00	5.00	100.00	0.37
M3G1	89.00	1.00	10.00	100.00	0.44
M4G1	84.00	1.00	15.00	100.00	0.47
M5G1	79.50	1.00	20.00	100.00	0.56
Group II: NPW ratio 3.0 wt. %					
M1G2	96.00	3.00	1.00	100.00	0.45
M2G2	92.00	3.00	5.00	100.00	0.51
M3G2	87.00	3.00	10.00	100.00	0.58
M4G2	82.00	3.00	15.00	100.00	0.60
M5G2	77.00	3.00	20.00	100.00	0.63

**Fig. 5:**
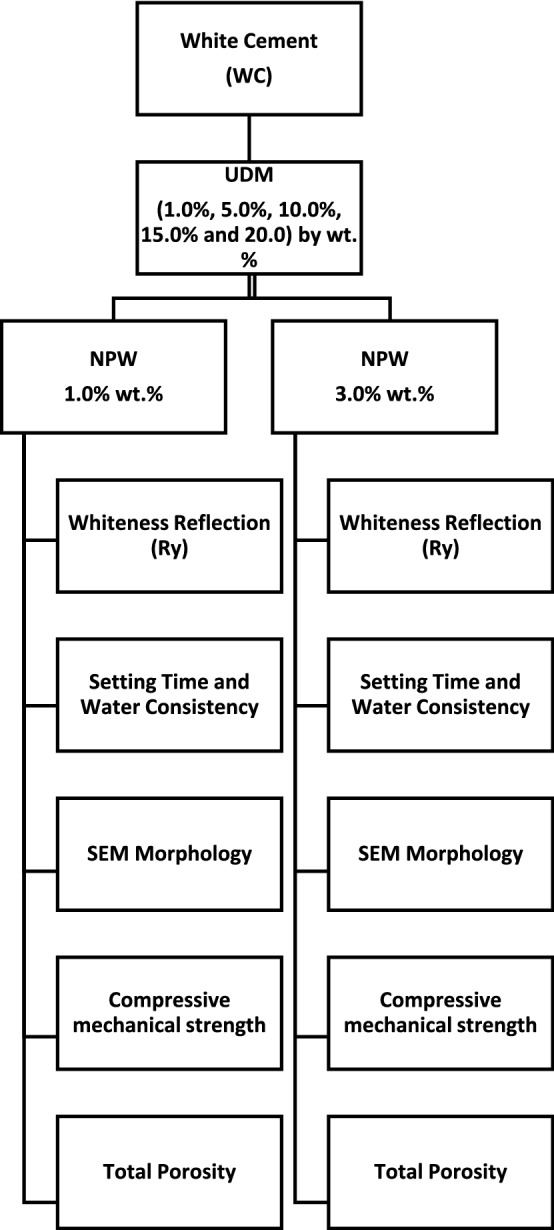
Scientific framework for instrumental and experimental program

After manual homo-process, the patches were cast in stainless steel molds with (25 × 25 × 25 mm) dimensions, and then, cubes set for hydration in 95 ± 5% actual humidity (RH) at ambient laboratory temperature. One day later, the cast prisms were de-molded and hydrated directly in tap fresh water for up to 28 days of hydration. The ASTM stander was the guide for our practical work as detailed elsewhere (Ariffin et al. 2015; ASTM 2016), which reflects the importance of (high RH) on the hydration process of the blends. In addition to investigating the workability of M_*x*_G_*x*_specimens, whiteness reflection, compressive strength, setting time, and porosity variations were estimated. Elerpho French apparatus was used to detect the whiteness reflection (Ry) comply with DIN 5033 specs (NORM 1992). Setting times and expansion (soundness) of M_*x*_G_*x*_-composites’ cement pastes were measured using Vicate and Le-Chatelier apparatus, respectively, based on ASTM C191 and ASTM C88, respectively (ASTM C191 2013; ASTM 2013). The compressive mechanical strength (CS) was triply performed according to ASTM C109M (Standard 2000), using a 5.00 ton load (e.g., Shemizitu German machine test) with a high loading rate of 20.00 kg min^−1^. Solidification of the prisms, e.g., porosity percentage, was conducted from porosimeter (Pore IV 9500), using mercury intrusion data (Standard 2000). According to records, the baselines for the pore sizes (macro-pores large than 3500 nm, micro-pores in 0–15 nm, while meso-pores in range between 15 and 3500 nm) were measured in this work. The residual specimen was carefully stored for XRD, TEM, and SEM analysis. The scientific schematic framework for the experimental program is plotted in Fig. 5.

### Instrumental analysis

Detailed chemical composition analysis for WC and UDW was performed using XRF (ARL 9900, Panalytical). The morphology of the specimens was determined with (FEI Company, The Netherlands), “an energy dissipation X-ray analyzer”. The transmission electron microscopy (TEM) instrument reported that the effective particle size for NPW is 56.93 and 81.14 nm. This indicated the NPW in nanosized powder, as seen in Fig. 4, which a suitable particle size for meso-pores of M_*x*_G_*x*_ matrix.

## Results and discussion for Group I

### Whiteness reflection (Ry)

**Fig. 6:**
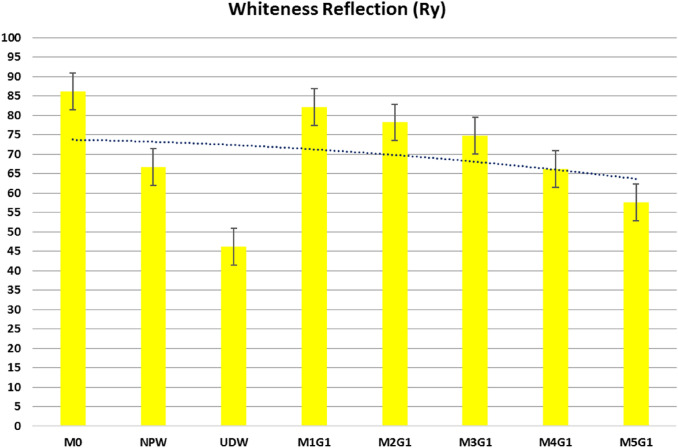
Whiteness Reflection (Ry) profile of proposed blends for Group I

Whiteness reflection (Ry) is one of the major indicators of white cement quality, so replacement will affect badly on WC reflection profile on the 3-axis (Rx = 86.24, Ry = 86.2, Rz = 80.59) (NORM 1992). Hinter l (Hl ≥ 80.0%) is the key perimeter for the whiteness intensity, while Hinter a (Ha ≤ -5.0) is the reflection of green and Hinter b (Hb) is the reflection of tallow color on Elerpho apparatus. Both NPW and UDW have a low degree of whiteness under Elerpho apparatus. Pale yellow color for NPW (Rx = 66.73, Ry = 66.70, Rz = 62.36) combined with too low whiteness color (Rx = 46.22, Ry = 46.20, Rz = 43.19) for UDW decreases M_x_G_x_ composites, as shown in Table 3. M_*x*_G_*x*_ composites pastes have the following order: M0 ˃ M1G1 ˃ M2G1 ˃ M3G1 ˃ M4G1 ˃ M5G1, as shown in Fig. 6. In addition, low Ha and Hb for NPW (Ha = − 1.78, Hb = 3.72) and UDW (Ha = − 1.48, Hb = 3.10) decrease the Hl for the blends as decreasing the green color and increasing in yellow color content. M1G1 shows better Hl intensity (Ha = 90.69), and this may be attributed to the equal portion 1.0% of both replacement and neutralized the Ry color (Ry = 82.20). High replacement of UDW decreased shapely the Ha for M5G1 paste (Hl = 75.88) with high content of yellowish color and poor green content.

**Table 3:** Whiteness reflection (Ry) of proposed blends for Group I

Pastes	Rx	Ry	Rz	Hl	Ha	Hb
M0	86.24	86.20	80.59	92.84	− 2.02	4.23
NPW	66.73	66.70	62.36	81.67	− 1.78	3.72
UDW	46.22	46.20	43.19	67.97	− 1.48	3.10
M1G1	82.24	82.20	76.85	90.66	− 1.97	4.13
M2G1	78.24	78.20	73.11	88.43	− 1.93	4.03
M3G1	74.85	74.82	69.95	86.50	− 1.88	3.94
M4G1	66.23	66.20	61.89	81.36	− 1.77	3.70
M5G1	57.61	57.58	53.83	75.88	− 1.65	3.46

### Setting time and water consistency

**Fig. 7:**
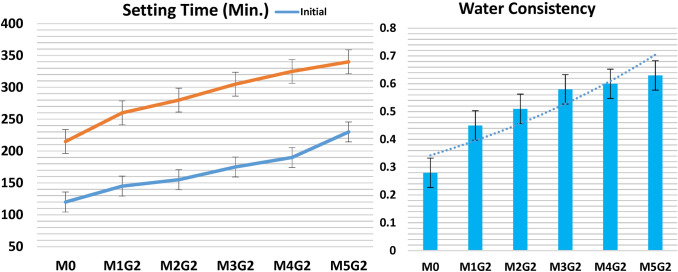
Setting time and water constancy of profile of proposed blends for Group I

Increasing the consistency of the water extends the initial and final setting time of the white cement composites. NPW acts as an inert filler, while UDW is a positive hydraulic filler. The setting time for the M_*x*_G_*x*_ blends—group I—has the following order: M0 ˂ M1G1 ˂ M2G1 ˂ M3G1 ˂ M4G1 ˂ M5T1, as shown in Fig. 7. Replacement of clinker/cement content by any filler enlarges the cement setting period, so hydraulic fillers are preferable additives as they form calcium-aluminate and calcium-sulfoaluminate during the pre-hydration process and reduce acoustic emission behavior (Kurda et al. 2019; Abdelzaher and Awad 2022). M1G1 and M2G2 show good workability and almost the same water/cement ratio, and it may be attributed to the equal ratio of inert/hydraulic filler ratio for M1G1 mix, which regulates the blend setting, similarity comes from performance during setting process. In addition; hydration process increases the solidification of blends and reduces early cracking. In contrast, M5G1 blend reflected the lowest workability due to high replacement of inert/hydraulic filler ratio, although it was in the nanoscale. Increasing inert/hydraulic filler content has a lower behavior on cement hydration phases. A high surface area fills open pores in the WC matrix and increases water demand. The average for white cement water/cement ratio by weight is ~ 0.40 ± 0.03 shows suitable water consistency and hydration products (Standard 2005).

### SEM morphology

**Fig. 8:**
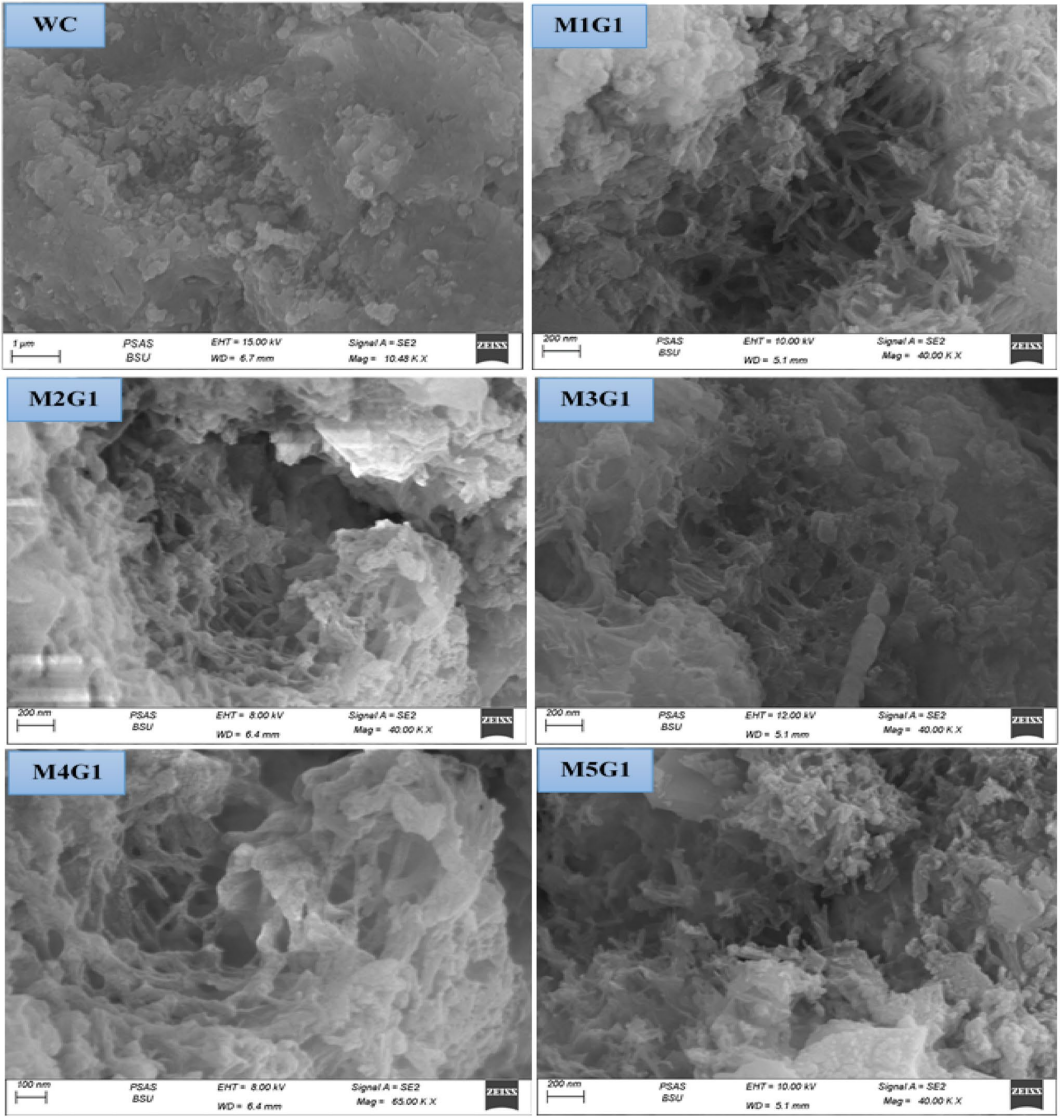
The morphology of M_*x*_G_*x*_-Group I paste composites

The WC microstructure is characterized by a less pore structure due to its ground to reach high surface area compared to the conventional OPC. Figure 8 shows the morphology of M_*x*_G_*x*_-Group I paste composites. Clearly, notice that the morphology of WC reflects good surface microstructure under the SEM apparatus with an arranged Skelton structure. M_*x*_G_*x*_-Group I paste composite morphologies are very interesting during SEM operation, as the appearance of linkage fibrous proves the presence of C–S–H gel and C–A–H phase (Abdelzaher and Shehata 2022; Abdelzaher 2021; Elkhouly et al. 2022). M1G1 (1.0% UDW) composite reports high fiber structure density leads to more solidification as UDW content, while reducing porosity content due to the NPW effect. The high silica content of UDW acts as active nuclei during the hydration process and promotes Tobermorite gel phase formation (Myers et al. 2013). As a substation of filler, increase the morphology changes badly at 1.0% wt. NPW filed the open pores without any hydraulic properties and UDW as substation increase, precipitated on the WC surface without extra hydraulic promotion. SEM reports that M4G1 (15.0% UDW) and M5G1 (20.0% UDW) composites have less fiber content and weak surface microstructure. We summarized the density of the fiber content quantitatively for M_*x*_G_*x*_-Group I paste composite in the following order: WC ˃ M1G1 ˃ M2G1 ˃ M3G1 ˃ M4G1 ˃ M5G1.

### Compressive mechanical strength (CMS)

**Fig. 9:**
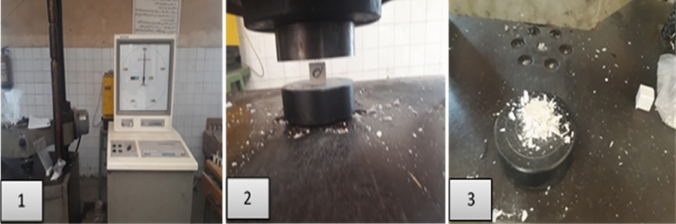
The compressive strength test on Shemizitu Machine

**Fig. 10:**
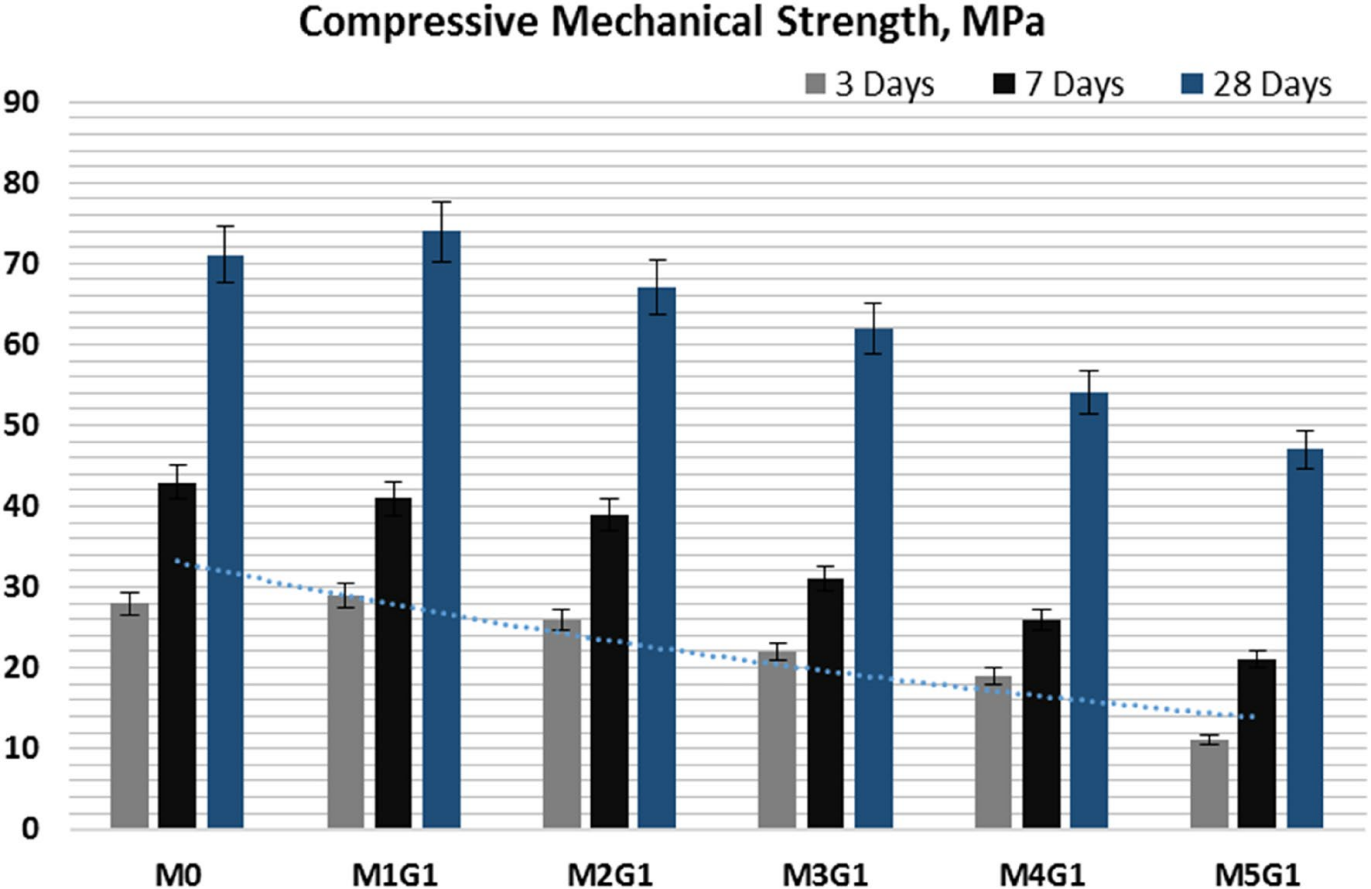
The compressive mechanical strength of M_*x*_G_*x*_-Group I paste composites

Eventually, compressive mechanical strength (CMS) is the key performance indicator for WC quality and workability. A low pore structure of WC reflects on CMS profile, as shown in Fig. 9, which illustrates visually the compressive strength test on Shemizitu German machine. Figure 10 shows the CMS of M_*x*_G_*x*_-Group I paste composites hydrated for 3, 7, and 28 days, respectively. Clearly, notice that the CMS varies with substitution level due to the replacement of hydraulic cementitious material by inert filler (NPW) and medium hydraulic filler (UDW). M1G1 paste shows beater CMS at early and late age of hydration, which may be attributed to the equal ratio from inert filler/medium hydraulic filler of 1:1% wt.%. Equal ratios make neutralization effect as high surface area of NPW and UDW fills the open pores of the WC microstructure, leading to solidification and hardness of M1G1 paste compared to the WC. Paste composites have the following order at an early age (3 days of hydration) in the CMS scale, e.g.: M1G1 (29 MPa) ˃ M0 (28 MPa) ˃ M2G1 (26 MPa) ˃ M3G1 (22 MPa) ˃ M4G1 (19 MPa) ˃ M5G1 (11 MPa) (Kim et al. 2018). At late age of hydration (curing for 28 days), M5G1 paste failed in CMS test and recorded 47 MPa, which was attributed to the high ratio of substitution from hydraulic cementitious material. The addition of UDW produces extra Alite clinker, but at limited ratios, as mentioned earlier that UDW can reach 8.0% wt.% substitution while saving the hydraulic properties (Costa and Ribeiro 2020); NPW promotes this limit to 10.0% wt.% substitution, e.g., M2G1 (67 MPa). Filling plastic waste into cement represents good room for improving the solid-waste recycling approach.

### Porosity

**Fig. 11:**
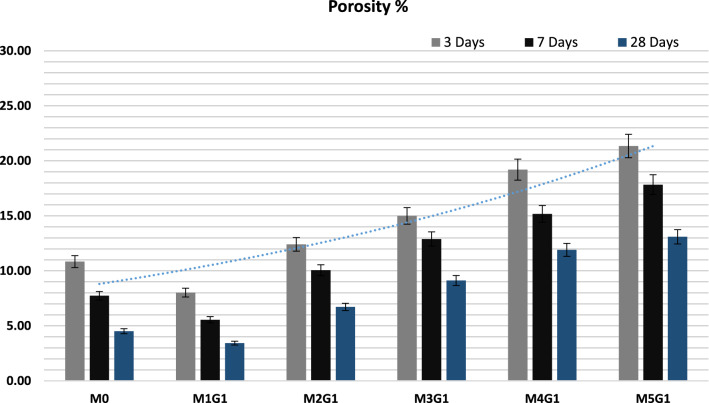
The porosity of M_*x*_G_*x*_-Group I paste composites

The pore volumes of MxGx-Group I paste composites after 3, 7, and 28 days of hydration are shown in Fig. 11. It is a water conjunction under certain conditions, and has a direct relation with compressive mechanical strength (Burwell et al. 1963). NPW combined with UDW affect positively on WC composite microstructure and rearrange the interior molecule structures, which lead to compact the surface area and increased solidification. Porosity decreases with curing age, and MxGx-Group I paste composites have the following order: M1G1 ˃ M0 ˃ M2G1 ˃ M3G1 ˃ M4G1 ˃ M5G1, which was also observed during SEM instrumentation analysis. It was clear that the porosity decreased by 13.7% for M1G1 as compared to M0 paste. At late hydration age (28 days of curing), porosity decreases sharply and recorded to less than 15.0%, e.g., M1G1 (4.02%) ˃ M0 (4.52%) ˃ M2G1 (7.72%) ˃ M3G1 (9.21%) ˃ M4G1 (11.1%) ˃ M5G1 (13.26%). Decreasing composites’ permeability delays the alkali (Cl^−^, Na, and K) ion penetration and reduces the alkali attack phenomena, e.g., corrosion, and this will lead to increase composites half-life time and cracking occurrence (Abdelzaher 2021).

## Results and discussion for Group II

### Whiteness reflection (Ry)

**Fig. 12:**
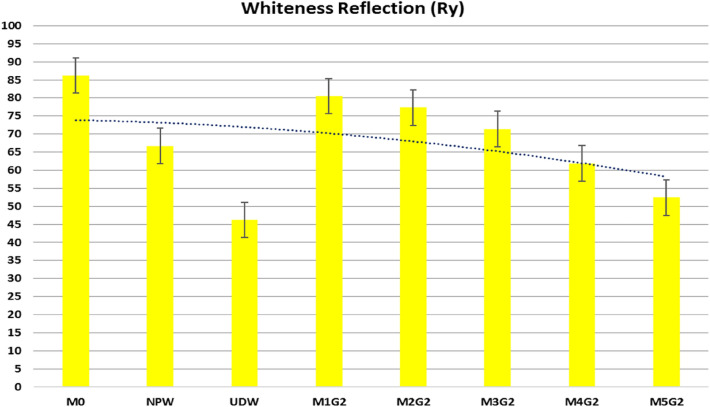
Whiteness Reflection (Ry) profile of proposed blends for Group II

It was clear that both NPW and UDW have a low degree of whiteness degree under Elerpho apparatus. Table 4 reports the Ry results for M_x_G_x_ composites pastes, which have the following order: M0 ˃ M1G2 ˃ M2G2 ˃ M3G2 ˃ M4G2 ˃ M5G2 as seen in Fig. 12. Further, low Ha and Hb for NPW (Ha = − 1.78, Hb = 3.72) and UDW (Ha = − 1.48, Hb = 3.10) respectively, decrease the Hl for the blends as decreasing the green color and increasing yellow color content. M1G2 shows better Hl intensity (Ha = 89.71), a low substation percentage compared to the color; Ry color (Ry = 80.48). High replacement of UDW decreased shapely the Ha for M5G2 paste (Hl = 72.39) with high content of yellowish color and poor green color content.

**Table 4:** Whiteness reflection (Ry) of proposed blends for Group II

Pastes	Rx	Ry	Rz	Hl	Ha	Hb
M0	86.24	86.20	80.59	92.84	− 2.02	4.23
NPW	66.73	66.70	62.36	81.67	− 1.78	3.72
UDW	46.22	46.20	43.19	67.97	− 1.48	3.10
M1G2	80.51	80.48	75.24	89.71	− 1.95	4.08
M2G2	77.37	77.34	72.31	87.94	− 1.91	4.00
M3G2	71.40	71.37	66.73	84.48	− 1.84	3.85
M4G2	61.92	61.89	57.86	78.67	− 1.71	3.58
M5G2	52.43	52.41	49.00	72.39	− 1.58	3.30

### Setting time and water consistency

**Fig. 13:**
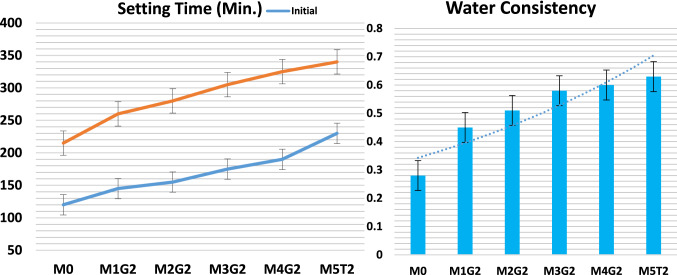
Setting time and water constancy of profile of proposed blends for Group II

Increasing substitution leads to increase in water/powder ratio. NPW acts as an inert filler, while UDW is a positive hydraulic filler. 3.0% wt.% NPW enlarges the consistency and elongates the setting time for the MxGx blends-group II, by default. Pastes have the following order: M0 ˂ M1G2 ˂ M2G2 ˂ M3G2 ˂ M4G2 ˂ M5T2, as shown in Fig. 13. Replacement of clinker/cement content by any filler enlarges the cement setting period, and the role of UDW as hydraulic fillers is a preferable additive as they form calcium-aluminate and calcium-sulfoaluminate during the pre-hydration process and reduce the acoustic emission behavior (Kurda et al. 2019; Abdelzaher and Awad 2022; Standard 2005). M1G2 showed good workability and almost the same water/cement ratio, which may be attributed to the low substitution ratio of inert/hydraulic filler ratio, which better regulates the blend setting behavior. Additionally, hydration process increases the solidification of blends and reduces early cracking. In contrast, M5G2 blend reflected the lowest workability due to a high replacement of inert/hydraulic filler ratio, although it was in the nanoscale. Increasing inert/hydraulic filler content has a lower behavior on cement hydration phases. A high surface area fills open pores in the WC matrix and increases water demand.

### SEM morphology

**Fig. 14:**
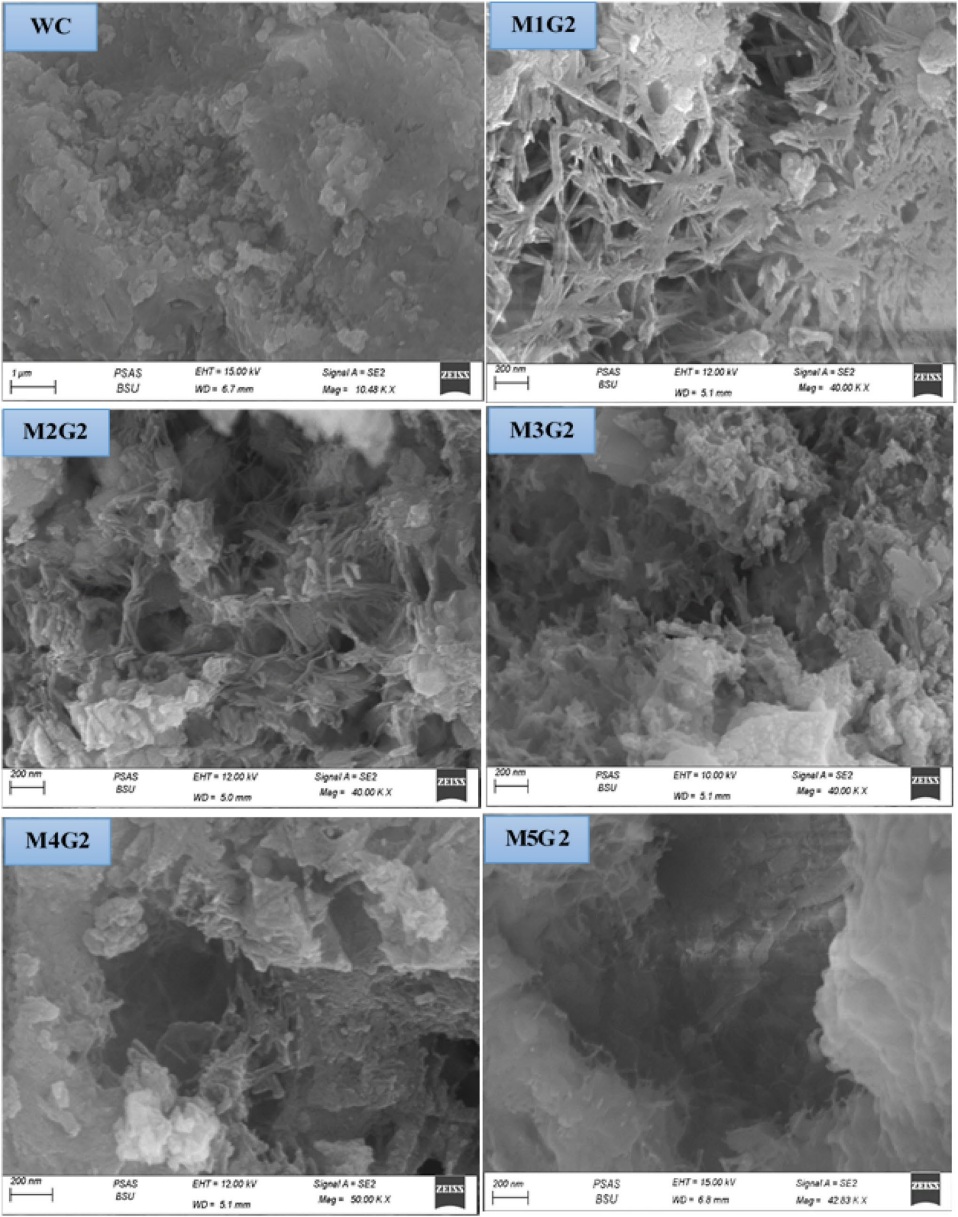
The morphology of M_*x*_G_*x*_-Group II paste composites

Figure 14 shows the morphology of M_x_G_x_-Group II paste composites. Clearly, notice that the morphology of WC reflects good surface microstructure under the SEM apparatus with an arranged Skelton structure. M_*x*_G_*x*_-Group II paste composite morphologies are varied during SEM operation, as the appearance of small amounts of linkage fibrous proves the presence of C–S–H gel and C–A–H phase (Abdelzaher and Shehata 2022; Abdelzaher 2021; Elkhouly et al. 2022). M1G1 (3.0% UDW) composite reports that low fiber structure density leads to less solidification than the WC, as high content of UDW, while reducing porosity content due to the NPW effect. As a substation of filler, increase the morphology changes badly at 3.0% wt. NPW filed the open pores without any hydraulic properties and UDW as substation increase, precipitated on the WC surface without extra hydraulic promotion. SEM reports that M3G2 (10.0% UDW), M4G2 (15.0% UDW), and M5G1 (20.0% UDW) composites have less fiber content and weak surface microstructure. We summarized the density of the fiber content quantitatively for M_x_G_x_-Group II paste composite in the following order: WC ˃ M1G2 ˃ M2G2 ˃ M3G2 ˃ M4G2 ˃ M5G2.

### Compressive mechanical strength (CMS)

**Fig. 15:**
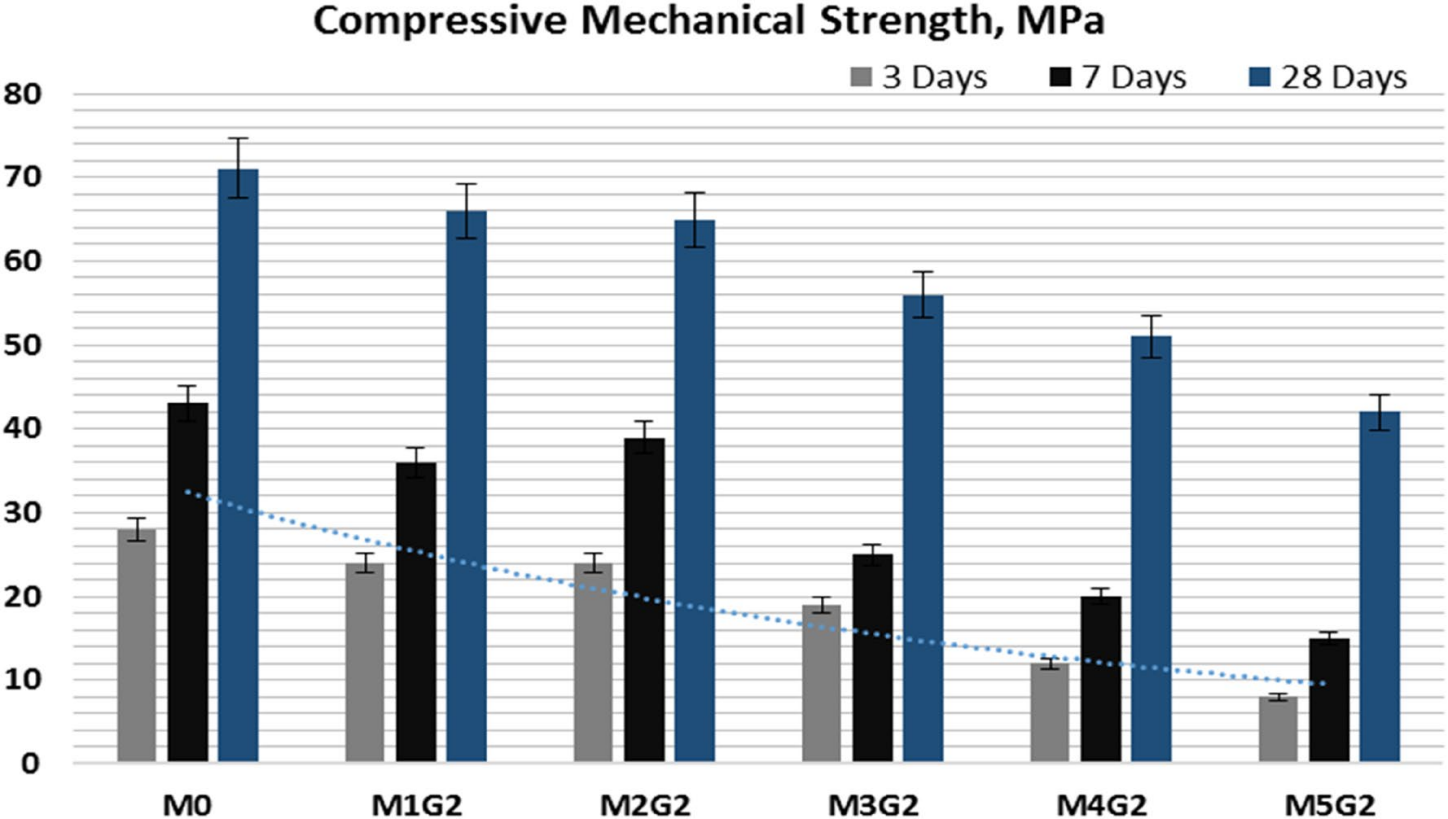
The compressive mechanical strength of M_*x*_G_*x*_-Group II paste composites

Figure 15 shows the CMS of M_*x*_G_*x*_-Group II paste composites hydrated for 3, 7, and 28 days, respectively. Clearly, notice that the CMS varies with substitution level due to the replacement of hydraulic cementitious material by inert filler (NPW) and medium hydraulic filler (UDW). NPW 3.0% wt. % showed a decrement in CMS at early and late age of hydration, which may be attributed to the high ratio from inert filler and medium hydraulic filler. In addition, the high surface area of NPW and UDW fills the open pores of the WC microstructure, leading to solidification and hardness of M_*x*_G_*x*_-Group II paste composites but lower the control sample. Paste composites have the following order at an early age (3 days of hydration) the CMS scale, e.g., M0 (28 MPa) ˃ M1G1 (24 MPa) = M2G1 (24 MPa) ˃ M3G1 (19 MPa) ˃ M4G1 (12 MPa) ˃ M5G1 (8 MPa). At late age of hydration (curing for 28 days), both M4G1 and M5G1 pastes failed in the CMS test and recorded 51 and 42 MPa, respectively, which was attributed to the high ratio of substitution from hydraulic cementitious material. The addition of UDW produces extra Alite clinker, but at limited ratios, as mentioned earlier that UDW can reach 8.0% wt. % substitution while saving the hydraulic properties (Kim et al. 2018).

### Porosity

**Fig. 16:**
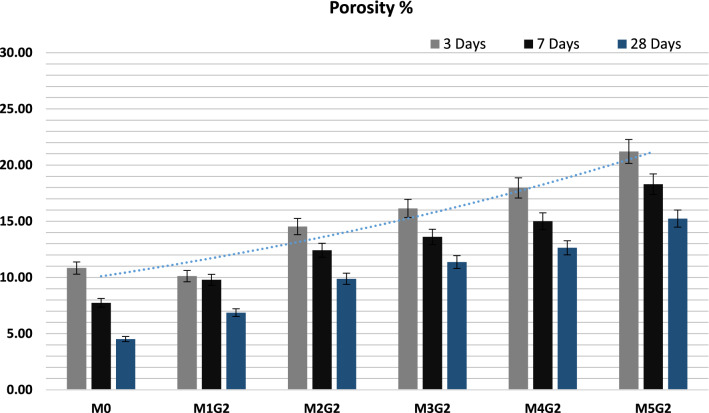
The porosity of M_*x*_G_*x*_-Group II paste composites

The pore volumes of M_*x*_G_*x*_-Group II paste composites after 3, 7, and 28 days of hydration are shown in Fig. 16. Eventually, NPW combined with UDW affect negatively on M_*x*_G_*x*_-Group II paste composites’ microstructure and rearrange the interior molecule structures, lead to weakness the surface area and decrease solidification. Porosity decreases with curing age, M_*x*_G_*x*_-Group II paste composites have the following order: M0 ˃ M1G2 ˃ M2G2 ˃ M3G2 ˃ M4G2 ˃ M5G2, which was also observed during CMS measurements. It was clear that the porosity sharply decreased by 27.2% for M1G1 as compared to M0 paste. At late hydration age (28 days of curing), porosity decreases sharply and recorded to less than 22.0%, e.g.: M0 (4.52%) ˃ M1G1 (6.87%) ˃ M2G1 (9.88%) ˃ M3G1 (11.37%) ˃ M4G1 (12.64%) ˃ M5G1 (15.24%). By default, decreasing composites permeability delays the alkali (Cl^−^, Na, and K) ion penetration and reduces the alkali attack phenomena, e.g., corrosion, this will lead to increase composites half-life time and cracking occurrence (Burwell et al. 1963).

## Conclusion

Solid-waste recycling is a major challenge nowadays. Reaching sustainability in raw material resources is the SDGs for industry, innovation, and infrastructure. The current practical investigation based on the 2050 vision is to reduce raw material consumption and CO_2_ emissions. NPW is an inert filler that can reduce the plastic waste dangerous worldwide and open room of recycling thesis waste in one of the most material consumption in the world, e.g., white cement industry. Two composite groups were proposed, with 1.0% and 3.0% fixed wt.% NPW incorporated with UDW from 1.0 to 20.0% (as substation). MIG1 (1.0% NPW + 1.0% UDW), (M2G1 (1.0% NPW + 5.0% UDW) and M1G2 (3.0% NPW + 1.0% UDW) showed better workability, whiteness reflection (Ry), and microstructure. In addition, NPW filled WC open pores enhance the physicomechanical features and reduce alkali leaching, improving sulfate attack properties. It recommend that until 3% of NPW can be applicable in the cement sector with additional physicomechanical features than the neat white cement.

## Data Availability

Not applicable.
